# Experimental realization of a Fresnel hologram as a super spectral resolution optical element

**DOI:** 10.1038/s41598-021-99955-w

**Published:** 2021-10-21

**Authors:** Mei-Li Hsieh, Thomas D. Ditto, Yi-Wen Lee, Shiuan-Huei Lin, Heidi J. Newberg, Shawn-Yu Lin

**Affiliations:** 1 The Department of Physics, Applied Physics and Astronomy, Rensselaer Polytechnic Institute, Troy, NY 12180 USA; 2 Department of Photonics, National Yang Ming Chiao Tung University, Hsinchu, Taiwan; 3 3DeWitt LLC, P.O. Box 10, Ancramdale, NY 12503-0010 USA; 4 Department of Electrophysics, National Yang Ming Chiao Tung University, Hsinchu, Taiwan; 5 College of Photonics, National Yang Ming Chiao Tung University, Hsinchu, Taiwan

**Keywords:** Applied optics, Optical physics, Techniques and instrumentation

## Abstract

A highly dispersive, diffractive optical element is designed and realized for an extremely high spectral resolution spectroscopy for exoplanet telescope application. Our design uses an annular Fresnel hologram to transform incident starlight directly into a spectrogram. The recording of the hologram is accomplished using two spherical waves of different radius of curvature. The resultant hologram consists of an annular grating structure with a gradually shrinking period as a function of increasing radius. The variable period not only could bring the incoming star-light into focus, but also exhibits a large on-axis chromatic behavior. We demonstrate a dispersion of wavelength 430–700 nm over 190 mm on-axis distance, leading to a super fine spectral resolution 0.0266 nm at wavelength 515 nm for a detector size of 20 µm.

## Introduction

**Figure 1:**
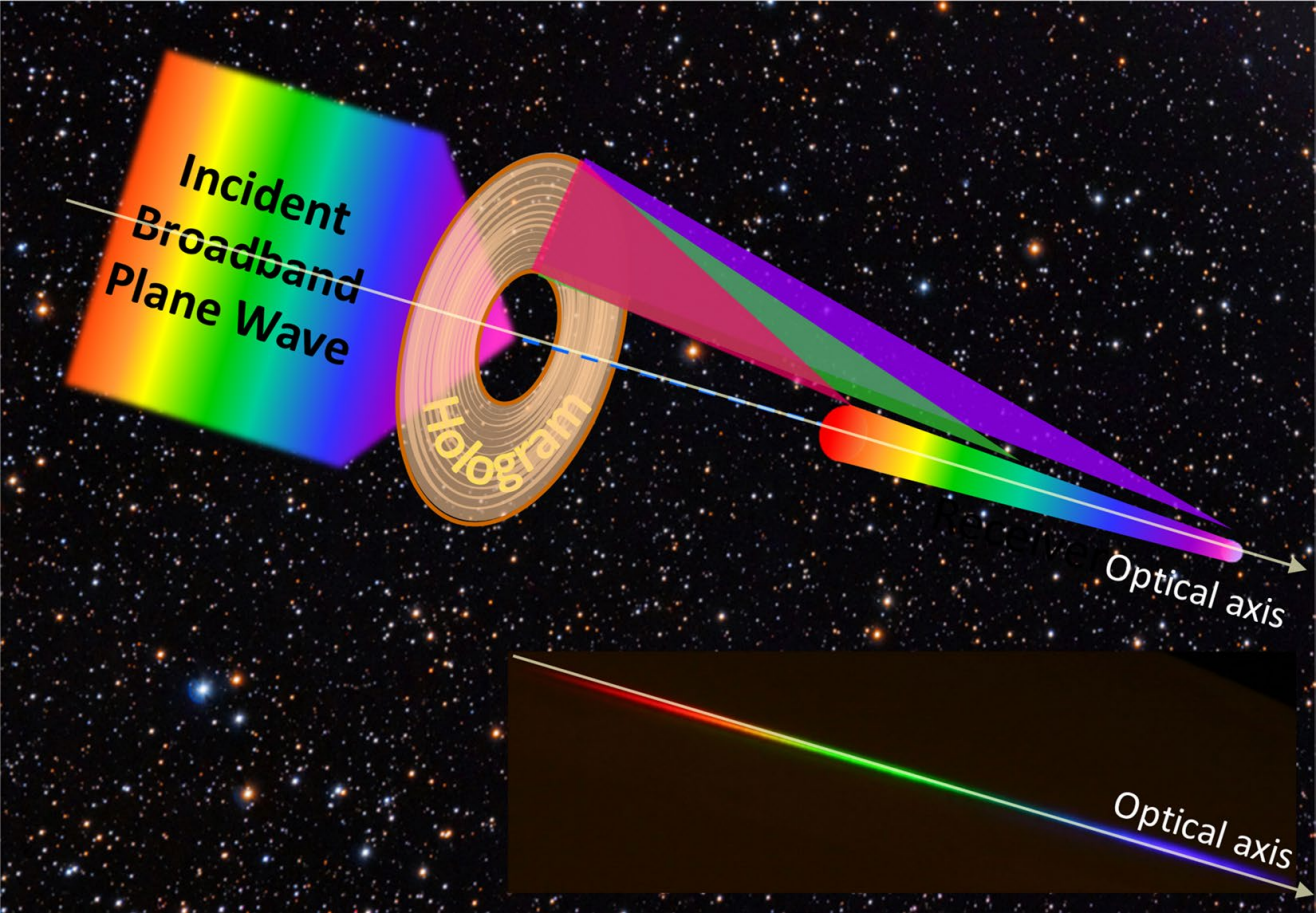
A schematic diagram of the dual use exoplanet telescope (DUET) based on a Fresnel hologram. (Inset) The photo shows a diffracted image of sunlight when passing through a Fresnel hologram.

For the past two decades, the phenomena of extreme light guiding, bending and dispersing based on the principle of diffraction has attracted much attention. Examples are sharp angle light-bending at one wavelength by a photonic crystal structure^1^, ultra sensitive light-dispersion in a photonic crystal structure^2^ and 90-degree light-bending in a volume hologram^3,4^. Furthermore, diffractive optical elements (DOEs) can achieve more complex functionalities than traditional refractive optical elements, such as lenses, prisms or aspheres, could. Also, DOE is much lighter in their mass density. Hence, DOEs offer an appealing solution for the construction of next-generation, ultra-large > 20 m space telescope primaries^5–10^. However, diffractive optical elements may produce a large chromatism when illuminated with a broad-bandwidth white light source. Nonetheless, the high chromatism of a DOE may be advantageous for spectroscopic applications. As an example, a novel telescope, Dual Use Exoplanet Telescope (DUET)^11^, was designed to directly analyze spectra of an exoplanet with extremely high spectral resolution based on diffractive optics. DUET can simultaneously perform high resolution spectroscopy and has a low areal mass for space exploration. Figure 1 shows a schematic diagram of such an exoplanet telescope. The main component of the telescope is an annulus gossamer membrane holographic primary objective with large collection area, yet its mass and stowage allow it to be delivered on a single lifter. In the inset, we show a photo of a diffracted image of sunlight when passing through our Fresnel hologram objective.

In this paper, we show a successful design and construction of a Fresnel hologram to focus light in the transverse direction (x–y plane) and disperse it in a linear fashion along the optical axis (z-axis). Previously, a holographic optical element (HOE) has been proposed to function as lenses for optical imaging purpose and was modeled using ray tracing method^12^. In this study, our Fresnel hologram is designed to function not only as a focusing but also a dispersive element along an optical axis. Additionally, the computational method we used is based on wave-optics principle. While typical FZP (Fresnel Zone Plate)^13^ is used for beam focusing of a specific wavelength, our hologram is designed to dispersed broad wavelengths over a long distance on axis. This Fresnel hologram constitutes the primary diffractive optics in a DUET system^10,11^. The main function of the Fresnel hologram is to collect the collimated star-light source and focus it into the detector. When a broad-band light source passes through an annulus diffractive optic, the incident light with different wavelengths will be focused into different positions along the optical axis. It is called dispersive behavior and can be applied for high resolution spectroscopy applications. In this paper, we will focus our effort on designing the annulus Fresnel hologram, realizing this hologram and demonstrating its unusually large chromatic behavior.

## Design and simulation results of the Fresnel hologram

**Figure 2:**
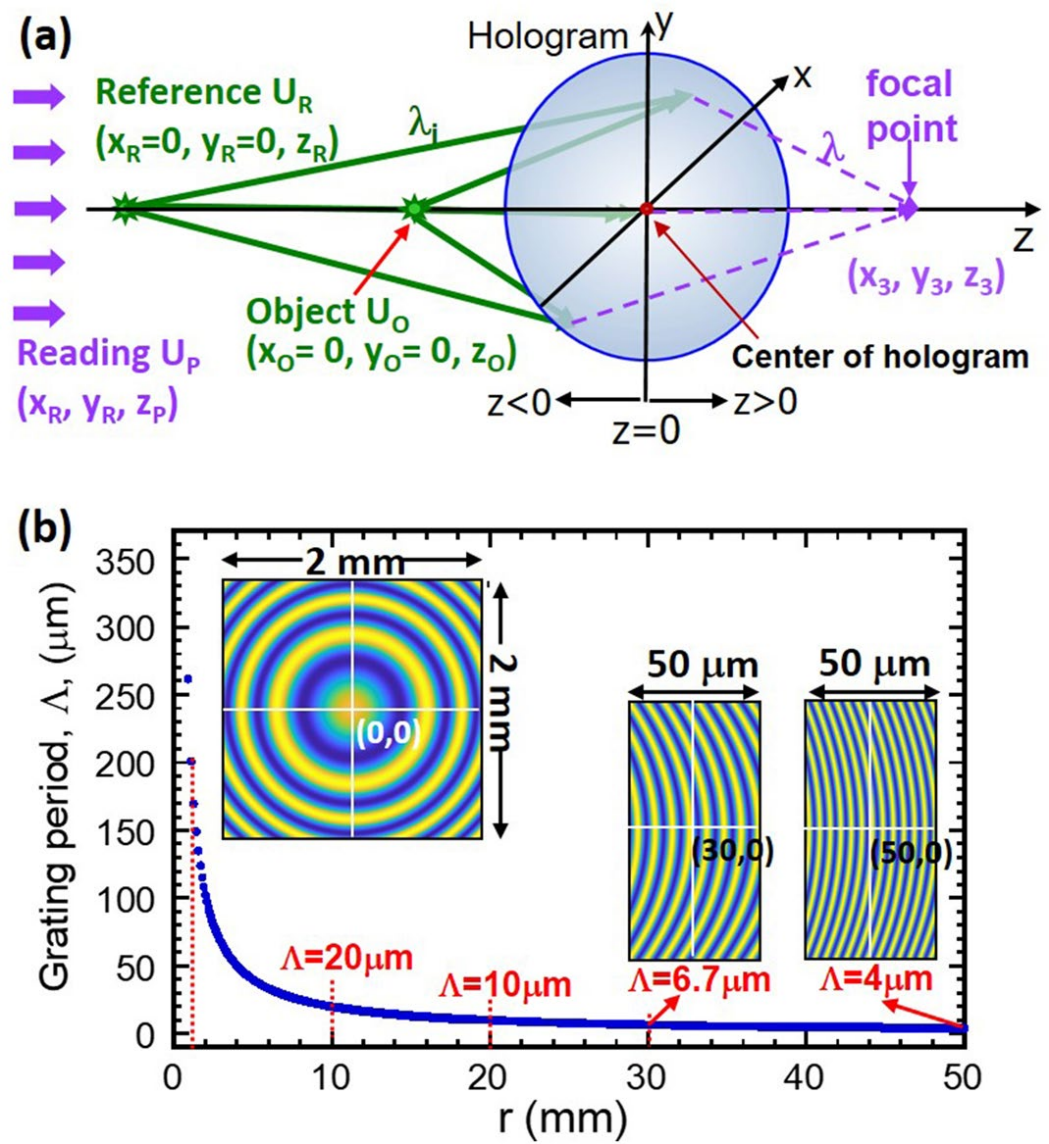
Experimental recording of the Fresnel hologram. (**a**) A schematic of the recording optical system using a diode-pumped-solid-state laser at *λ* = 515 nm. The reference and object are both point sources, emitting spherical waves for hologram recording. (**b**) A plot of the resulting grating pitch v.s. radial distance (*r*) away from the center of the hologram. (Inset) Images of intensity distribution of the interference pattern at three different positions of the hologram, i.e. (x, y) = (0, 0), (30 mm, 0) and (50 mm, 0).

Our Fresnel hologram is based on the interference of two spherical waves, emerging from point sources *U*_R_ (Reference) and *U*_O_ (Object), on a hologram film located at *z* = 0 plane. Note that a typical Fresnel zone plate is produced using a plane-wave and a spherical wave, which may be viewed as a special case of our two spherical-waves configuration. Figure 2a shows a schematic diagram of the two-wave recording geometry. The reconstruction of the hologram is achieved by placing another point source *U*_P_ at the same side of *U*_R_ and *U*_O_ (*z* < 0) and then searching for point image formation at the other side of the hologram, i.e. *z* > 0. The recording wavelength, λ_i_, of *U*_R_ and *U*_O_ may be different from the reading one of *U*_P_, *λ*. When the propagating waves from *U*_R_, *U*_O_ and *U*_P_ satisfy the paraxial approximation^14^, formation of a point image can occur near or on the optical axis. Also, noted that the focal length of the point image is expected to be λ-dependent.

In this paper, we consider two special conditions: (1) both *U*_R_ and *U*_O_ are placed on the *z*-axis, leading to an on-axis hologram with cylindrical symmetry and annular shape of the interference pattern; (2) *U*_P_ is placed quasi-infinitely away from the hologram, so it has a planar wave-front. Under this configuration, our hologram can image (or concentrate) a collimated, single *λ* beam onto a point on the axis. It can also image a broadband, collimated beam onto a series of points along the axis. Traditionally, when a broadband radiation of a given intensity is dispersed in space, it gains angular spread of *λ* but at the expense of losing its intensity flux. On the contrary, for our Fresnel hologram, a broadband radiation is dispersed along the optical axis but each wavelength of light is focus on its own focal point. This is an important feature of our hologram, leading to both super λ-resolution and super sensitive detection for an exoplanet telescope.

We now present a brief derivation of the theoretical framework of image formation. A more detail account of it is discussed in the Method section. From Fig. 2a, the reference wave *U*_R_ is on (*x*_R_, *y*_R_, *z*_R_) and the objective wave *U*_O_ on (*x*_O_, *y*_O_, *z*_O_). The two recording waves propagate along the optical axis and interfere at *z* = 0 plate. The intensity distribution of the interference pattern of the two spherical waves can be written as:

1 $$I\left( {x,y,z} \right) = \left| {U_{R} + U{}_{O}} \right|^{2} \approx \left| {A_{{\text{R}}} \frac{{e^{{ik\sqrt {(x - x_{R} )^{2} + (y - y_{R} )^{2} + \left( {z - z_{R} } \right)^{2} } }} }}{{\left| {z_{R} } \right|}} + A_{{\text{O}}} \frac{{e^{{ik\sqrt {(x - x_{o} )^{2} + (y - y_{o} )^{2} + \left( {z - z_{O} } \right)^{2} } }} }}{{\left| {z_{O} } \right|}}} \right|^{2}$$

Here, *A*_R_ and *A*_O_ are the amplitude of the reference and objective beams, respectively. *k* is the recording beam wave vector, *k* = 2*π*/*λ* and *λ*_i_ is its wavelength. Under the Paraxial approximation. *U*_R_ and *U*_O_ may be re-written as :

2 $$\begin{aligned} U_{R} & = \left( {A_{R} /z_{R} } \right)e^{{ik\sqrt {(x - x_{R} )^{2} + (y - y_{R} )^{2} + (z - z_{R} )^{2} } }} \approx \left( {A_{R} /z_{R} } \right)e^{{ik\phi_{R} }} \\ U_{O} & = \left( {A_{O} /z_{O} } \right)e^{{ik\sqrt {(x - x_{O} )^{2} + (y - y_{O} )^{2} + (z - z_{O} )^{2} } }} \approx \left( {A_{O} /z_{O} } \right)e^{{ik\phi_{O} }} \\ \phi_{R} & \equiv - \frac{\pi }{{\lambda_{i} z_{R} }}\left( {x^{2} + y^{2} - 2xx_{R} - 2yy_{R} } \right) \\ \phi_{O} & \equiv - \frac{\pi }{{\lambda_{i} z_{O} }}\left( {x^{2} + y^{2} - 2xx_{O} - 2yy_{O} } \right) \\ \end{aligned}$$

To simplify Eq. (1) and also to achieve the maximum contrast of the interference pattern, the amplitude of the reference and objective beams are set to *A*_R_/|*z*_R_| = *A*_O_/|*z*_O_| = 1. Thus, the intensity distribution of the interference pattern becomes:

3 $$I\left( {x,y,z} \right) = \left| {e^{{ik\sqrt {x^{2} + y^{2} + \left( {z - z_{R} } \right)^{2} } }} + e^{{ik\sqrt {x^{2} + y^{2} + \left( {z - z_{O} } \right)^{2} } }} } \right|^{2}$$

To illustrate the annular feature of the hologram, we compute the intensity distribution of the interference pattern using Eq. (3). The results are shown in the inset of Fig. 2b. Here, *λ*_i_ = 515 nm and the two spherical waves are located at *z*_R_ = − 72 cm and *z*_O_ = − 25.2 cm, respectively. The interference pattern at the origin (*x*, *y*) = (0, 0) is shown on the upper left over an area 2 × 2 mm^2^. The blue color represents the minimum intensity and the yellow the maximum intensity. It exhibits an annular-like grating structure, consistent with the axial symmetry of the recording and reading configuration. Here the radius of first blue circle is 380 μm and the grating pitch between the first and second blue circle is Λ = 260 μm. Additionally, the interference patterns at (*x*, *y*) = (30 mm, 0) and (50 mm, 0) are shown on the lower right corner over an area 50 × 100 µm^2^. They both display a shorter grating pitch. The grating pitch is reduced to Λ = 6.7 µm at (30 mm, 0) and further reduced to Λ = 4 µm at (50 mm, 0). Theoretically, the grating pitch of the intensity distribution pattern is a function of the radial position *r* = (*x*^2^ + *y*^2^)^1/2^. Figure 2b plots the grating pitch as a function *r* as the blue color curve. The grating pitch decreases rapidly as *r* is increased. For instance, grating pitch decreases from Λ = 201, 20, 6.7 to 4 µm as the radius is increased from *r* = 1, 10, 30 to 50 mm, respectively. Note that the decreasing Λ from the origin to the outer regions serves to bring the incoming plane wave to focus much like the traditional Fresnel zone plate^13^. This provides for a mechanism for the hologram to strongly concentrate light to a “point” bounded only by the diffraction limit.

The focal-point of our hologram, i.e. (*x*_3_, *y*_3_, *z*_3_), may be derived analytically as well. Again, its full derivation is discussed in the Method section. Briefly, the derivation principle is to match the phase factor from the object (*ϕ*_O_) and recording beam *ϕ*_*R*_ to the phase factor of the diffracted beam *ϕ*_3_. As a result, the focal point (*x*_3_, *y*_3_, *z*_3_) of the image is a function of *z*_R_, *z*_O_, *λ*, *λ*_*i*_, and given by:

4 $$\begin{aligned} x_{3} & = y_{3} = 0; \\ \frac{1}{{z_{3} }} & = \left( {\frac{1}{{z_{R} }} - \frac{1}{{z_{O} }}} \right)\frac{\lambda }{{\lambda_{i} }} \, \;\;\;or\;\;\; \, z_{3} \lambda = \left( {\frac{1}{{z_{R} }} - \frac{1}{{z_{O} }}} \right)^{ - 1} \lambda_{i} \\ \end{aligned}$$

This derivation shows that, for our hologram design, the image is located on-axis (*x*_3_ = *y*_3_ = 0) and the product of (*z*_3_*λ*) is a constant. In our experimental case, when *z*_R_ = − 72 cm, *z*_O_ = − 25.2 cm, *λ*_i_ = 515 nm, (*z*_3_*λ*) = 19,966 cm-nm. Note that *z*_3_ is a positive number. This means that the diffracted output is focused onto the *z*_3_ position forming a real image. On contrary, if *z*_3_ is a negative number, the diffracted output becomes a divergent wave that originates from − |*z*_3_| position. Hence, both the recording parameters, *z*_R_ and *z*_O_, provides for a new design degree-of-freedom for producing either a convergent or divergent diffracted beam. The combinational use of two spherical waves also allow us to design a large value of *z*_3_ (when *z*_R_ ≅ *z*_O_), and thus can produce a highly dispersive optical element. The position *z*_3_ of the diffracted spherical wave, measured from the hologram plane, is called the focal-length of the hologram.

Because *z*_3_ is inversely proportional to the reading *λ*, our hologram has a significant chromatic dispersion property when illuminated with a broadband light source. When *λ* = *λ*_i_ = 515 nm, *z*_30_ = 38.8 cm and is the reference focal-length of the image. When $\lambda \ne \lambda_{i}$, the diffracted wave is focused onto a different position along *z*-axis such that $z_{3} \ne z_{30}$. For instance, when *λ* is varied from *λ* = 400 nm to 700 nm, the corresponding focal length is shifted from *z*_3_ = 49.9 cm to 28.5 cm. This result shows that the hologram can achieve a spectral dispersion of 400 nm to 700 nm wavelength over an on-axis distance of 21.4 cm. An important feature of our hologram is its ability to achieve a long tuning length for a super high spectral resolution. It originates from an unusual on-axis dispersive behavior of the Fresnel hologram.

**Figure 3:**
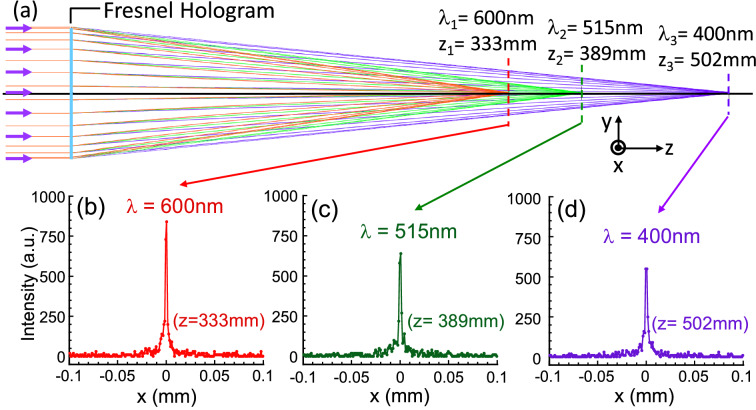
A simulation result of the reconstructed image at three different wavelength of light, λ_1_ = 600, λ_2_ = 515 and λ_3_ = 400 nm. (**a**) The effect of light focusing and dispersion by a Fresnel Hologram simulated by the ZEMAX software. (**b**–**d**) are the simulated intensity distribution of light versus x-axis of three different ls at their respective focal planes, z_1_ = 333, z_2_ = 389 and z_3_ = 520 mm, respectively.

To independently confirm the chromatic dispersion of our hologram, we use commercially available software, Zemax®^15^, to simulate the performance of our hologram. The simulated result is illustrated in Fig. 3. In this simulation, the hologram has a circular size with a diameter *D* = 10 cm. It is modeled in accordance with our recording parameters, i.e. the two point sources are at *z*_R_ = − 72 cm and *z*_O_ = − 25.2 cm and the recording *λ*_*i*_ = 515 nm. Also, its grating period varies as a function of r as shown in Fig. 2b. The illuminating light is chosen to be collimated and contains three different colors, i.e., *λ* = 600 nm (red), 515 nm (green) and 400 nm (blue). The result shows that the three colors of light are been focused onto three different spots along the *z*-axis. We obtain *z*_3_(*λ* = 600 nm) = 333 mm, *z*_3_(*λ* = 515 nm) = 389 mm, and *z*_3_(*λ* = 400 nm) = 500 mm. We found that the shift of the focal length versus λ is consistent with that predicted from Eq. (4). This result numerically demonstrates the unusually large chromatic dispersion produced by our hologram. Figure 3 also plots the intensity distribution of the three wavelengths at their respective focal plane and along the *x*-axis. The spatial intensity distributions for all three colors are sharp. We found the diameter of the focus points to be less than 5 µm. Although, we note that Zemax® is based on ray tracing method and the minimum focus spot should be set by the diffraction limit. Nonetheless, the intensity distribution plots illustrate the unusual light concentration capability of our hologram. This simulation shows that our hologram can achieve strong concentration of a single color and also on-axis stretching of multi-colors all at the same time.

## Experimental discussion

**Figure 4:**
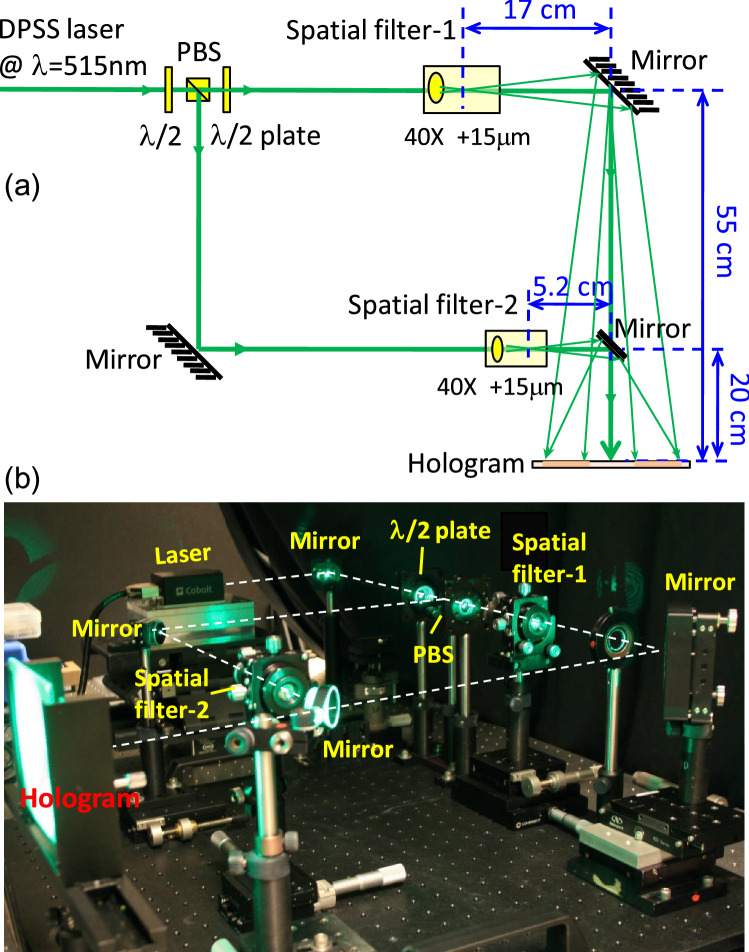
(**a**) A schematic diagram of the optical recording system for Fresnel Hologram. (**b**) A photo of the recording setup using a λ = 515 nm laser. The optical path of light is indicated by white dashed lines.

We have established an optical system to realize the Fresnel hologram and experimentally demonstrate the chromatic behavior of the hologram. Figure 4a shows a schematic diagram of the optical setup system. The laser we used for hologram recording is a collimated, diode-pumped-solid-state (DPSS) laser with a single longitudinal mode output. It emits light at *λ* = 515 nm and has a long coherent length of about one-meter. The laser light is first split into two beams, where one is the reference and the other the object beam. The half-wave (*λ*/2) plate is used to adjust the relative intensity of the reference and objective beams. Both beams are focused through 40X objective lenses and then passed through pinholes of 15 µm diameter. This procedure produces point light source and, therefore, the desirable propagating spherical waves. The pinholes for the reference and object beams are located at *z*_R_ = − 72 cm and *z*_O_ = − 25.2 cm away from the hologram plane, respectively. In order to record an on-axis hologram, an additional one-inch mirror is used to guide and align the object beam such that it propagates along the same optical axis as the reference beam. Finally, a Litiholo holographic film of 4" × 5" size is placed at the desired exposure position to record the hologram. Figure 4b shows a photo of the optical recording system for our Fresnel hologram. Note the size of a realizable hologram is limited by the power and finite coherent length of a laser. Using our current optical setup system, the hologram might be scaled up to about one-meter in diameter.

**Figure 5:**
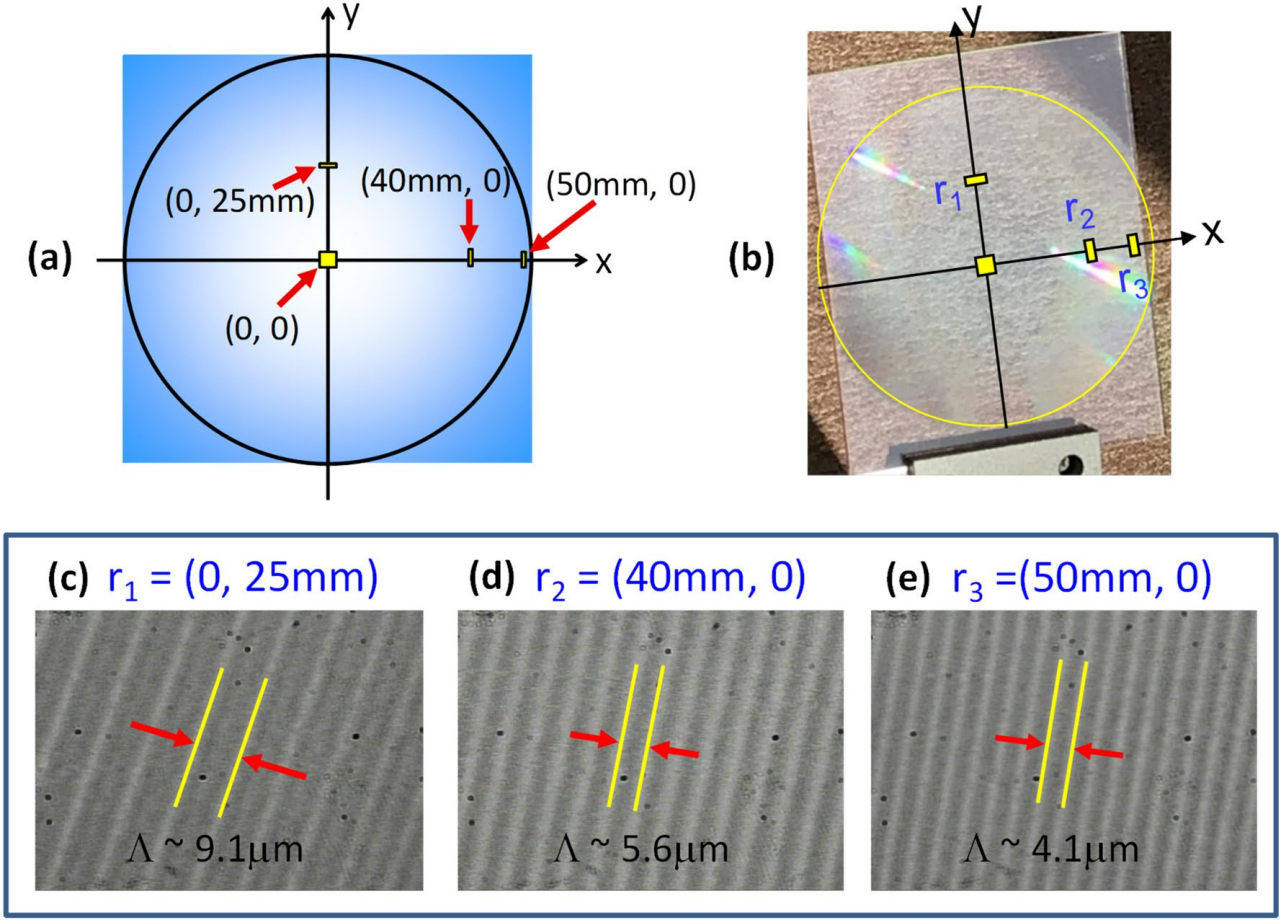
(**a**) A schematic of the hologram coordinate at z = 0 plane, where both the reference and object point sources are positioned along the z-axis. (**b**) A photo of the recorded 4 × 5 inch^2^ Fresnel hologram on a glass plate. (**c**–**e**) Optical images of the recorded grating structure taken at different points, r_1_, r_2_ and r_3_, on the Fresnel hologram. The grating pitch Λ becomes denser as the observation point is further away from the center.

Once the hologram is recorded, a high resolution microscope is used to verify if the resultant interference pattern follows the design prediction shown in Fig. 2b. Figure 5a shows a schematic drawing of the hologram and coordinates where microscope images were taken. Figure 5b shows a photo of the 4" × 5" hologram film after been exposed to ~ 40 mJ/cm^2^ recording energy. Figure 5c–e show microscope images of the hologram at point *r*_1_ = (0, 25 mm), *r*_2_ = (40 mm, 0) and *r*_3_ = (50 mm, 0), respectively. In all three images, clear grating structures were observed that illustrate a successful interference of the reference and object beams. The grating period is systematically reduced from Λ ~ 9.1, 5.6 to 4.1 µm as the coordinates radius is increased from *r* = 25, 40 to 50 mm, respectively. The decreasing trend of Λ toward outward region of the hologram is consistent with the trend predicted in Fig. 2b. It is noted that the theoretical simulation predicts a grating period of Λ ~ 8.0 and 4.0 µm at *r* = 25 and 50 mm, respectively. There are two reasons that might cause a slight discrepancy between the observed and predicted grating period. One is due to a non-ideal optical alignment between the two recording beams. For instance, from our calculation, if the two beams are not perfectly aligned along the same direction and off by 0.1°, it will result in a deviation of grating period by ~ 1.1 µm at *r* = 25 mm. Conversely, it may be stated that the upper bound of our alignment error is within 0.2°. The other is a possible measurement error of finding the exact central position of the almost transparent hologram under the microscope. Nonetheless, the observation of clear grating structures and the agreement of grating periods between experiment and theory throughout the hologram film indicate that we have successfully recorded a Fresnel hologram.

**Figure 6:**
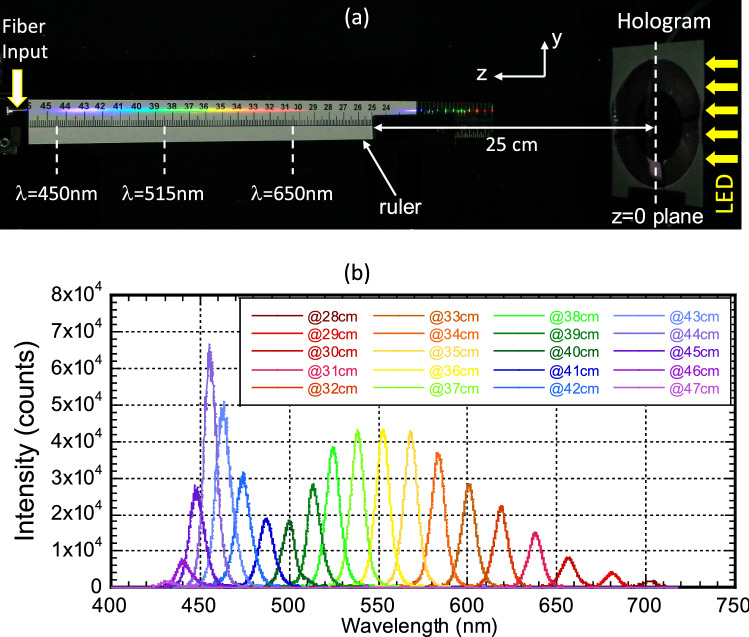
Reconstructed image and spectroscopic measurement of the Fresnel hologram. (**a**) A photo of the reconstructed image using a collimated LED white light. The ruler is placed near the optical axis, z-axis, to illustrate the chromatic behavior. (**b**) A series of spectral curves taken at different positions along the optical axis using an optical fiber of core diameter d = 400 µm.

The recorded hologram is expected to function simultaneously as a chromatic and a focusing element. Figure 6 experimentally illustrates the chromatic behavior of the hologram. A white LED light (covering *λ* = 450–650 nm spectral range) is used to illuminate the hologram. The LED light serves as a point source, which is expanded and collimated using a Cassegrain reflecting telescope^16^ in a reverse input configuration and normally incident onto the hologram. The transmitted wave is then diffracted and imaged onto different positions on the *z*-axis in accordance to their specific *λ*. Figure 6a shows a photo of the reconstructed image of the hologram. Firstly, the white LED light is well focused and dispersed along the optical axis, where a ruler is carefully placed near the axis. The finite spread of light along the *y*-direction is due to a slight mis-alignment of the ruler to the z-axis and also the scattering of light by the ruler. The actual spot size is much smaller and is defined by the diffraction limit of the hologram size, see Fig. 3b. Secondly, the white LED light is dispersed into a series of vivid colors, ranging from red, orange, yellow, green, blue and purple. The observation of bright color (i.e. strong intensity) for a dispersed light is in sharp contrast to those of typical dispersive elements, such as a prism, where light is dispersed, spread in all three dimensions and its intensity flux greatly weakened. Finally, consistent with the prediction of Eq. (8), the shorter wavelength (*λ*) light has a longer focal point (*z*_3_).

While the concentration capability of our hologram is characterized by a tight spatial extend of the focused spot in the transverse plane (*x*–*y*) shown in Fig. 3b, its chromatic behavior is quantified by a long spatial extend along the longitudinal axis (*z*-axis). Here we perform a spectroscopic study of the spectral resolution of the focused light at a series of focal points along the *z*-axis. A fiber coupled optical spectrometer (Ocean Optics USB4000) with a fiber core diameter of *d* = 400 µm is utilized to measure the spectrum. The fiber, shown in the far left of Fig. 6a, is to be translated along the *z*-axis to perform spectroscopic measurement. Figure 6b shows a series of measured spectra taken at a range of *z*-position from *z* = 28 cm to 47 cm. Firstly, the measured spectra show stronger intensities at *λ* ~ 455 nm (blue color) and *λ* ~ 550 nm (yellow) region. This is due to spectral distribution of the primary and secondary emission of the LED light used in our testing^17^. Secondly, the measured spectra displayed well-defined peak wavelength, which are shifted systematically toward longer-*λ* for shorter *z*. The full-width-half-maximum (FWHM) of the individual peak is Δ*λ*_FWHM_ ~ 7.5 nm at *λ* ~ 450 nm and increased by about two times to Δ*λ*_FWHM_ ~ 15 nm at *λ* ~ 650 nm. The relatively large FWHM is mainly due to the finite size of the fiber core diameter *d* = 400 µm. It does not represent the intrinsic spectral resolution of the hologram, which is to be addressed in a following paragraph.

**Figure 7:**
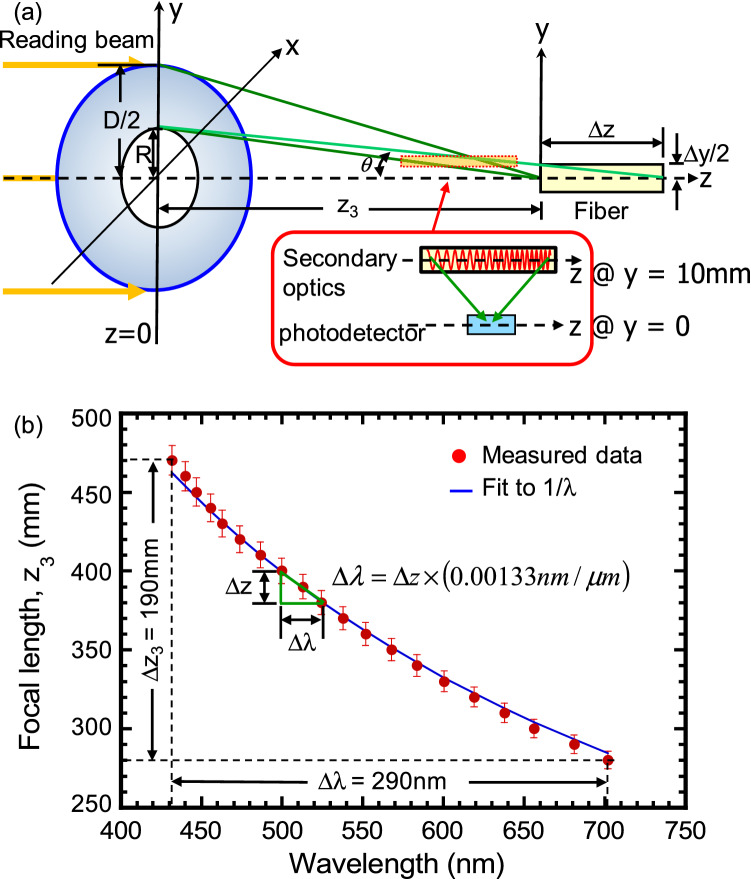
Spectra dispersion and resolution of Fresnel Hologram on the optical axis. (**a**) Reading geometry of the hologram and the image of a monochromatic light λ at its focal length *z*_3_. The detecting length Δ*z* and width Δ*y* are also indicated. (**b**) A summary plot of the measured focal length *z*_3_ versus reading *λ*. The red dots are experimental data with 2% error bar and the blue curve is a simulated result (*z*_O_ = − 25.2 cm, *z*_R_ = − 72 cm).

Thirdly, the measured peak is shifted from *λ* = 440 nm to 705 nm as the focal length is decreased from *z* = 470 mm to 280 mm. So, white light of Δ*λ* = 265 nm bandwidth is well dispersed into a spatial extend of Δ*z* = 190 mm. Figure 7b summarizes the measured (red dots) and predicted (blue curve) focal length as a function of λ. The measured focal length shows a decreasing trend as λ is increased. Recall from Eq. (4) that $z_{3} \lambda = z_{30} \lambda_{i}$, or

5 $$z_{3} \left( \lambda \right) = \left( {\frac{{z_{30} }}{\lambda }} \right)\lambda_{i}$$

where $z_{30} = \left( {{1 \mathord{\left/ {\vphantom {1 {z_{R} }}} \right. \kern-\nulldelimiterspace} {z_{R} }} - {1 \mathord{\left/ {\vphantom {1 {z_{o} }}} \right. \kern-\nulldelimiterspace} {z_{o} }}} \right)^{ - 1}$. So, from Eq. (5), the chromatic behavior, *z*_3_(*λ*), of our hologram is characterized by the pre-factor "*z*_30_". When "*z*_30_" is increased by recording design, the incoming starlight will be more dispersed and the hologram is more suited for achieving super-resolution purpose. When "*z*_30_" is reduced, the diffracted λ of the incoming starlight is more compressed (focused) along the *z*-axis and the hologram is more suited for high sensitivity application. Returning to experimental data, when the reading-*λ* equals the recording *λ*_i_ = 515 nm, *z*_3_ = *z*_30_ = 388 mm (the reference). The measured focal length value is *z*_30_ = 385 mm, which is in good agreement with the predicted result. Note also that *z*_3_ should follow the (1/*λ*) dependence. And, indeed, our measured focal length accurately reproduces the predicted values (the blue curve).

In the following, we discuss spectral resolution and starlight detection sensitivity of our Fresnel hologram. The spectral resolution Δ*λ* of our hologram is determined by the detection length Δ*z* of a detector of choice along the z-axis. Let us assume that Δ*z* << *z*_30_ = 388 mm and rewrite Eq. (5) in a differential form, we find: $\Delta \lambda \cong - \left( {\frac{\Delta z}{{z_{30} }}} \right)\left[ {\frac{{\lambda^{2} }}{{\lambda_{i} }}} \right]$. The formula indicates that the spectral resolution depends on the intrinsic property of the hologram (*λ*_*i*_*z*_30_), the detection length Δ*z*, as well as *λ*^2^. This explains our earlier observation in Fig. 6b that the spectral linewidth Δ*λ*_FWHM_ is wider for longer-*λ* peaks. Also, when *λ* is increased from 450 to 650 nm, the theory predicts a (650/450)^2^ ~ 2 × increase of linewidth which agrees with the observation shown in Fig. 5b. As a specific example, when *λ* = *λ*_i_ = 515 nm and *z*_30_ = 388 mm, we have $\Delta \lambda \cong - \Delta z \times \left( {{{\lambda_{i} } \mathord{\left/ {\vphantom {{\lambda_{i} } {z_{30} }}} \right. \kern-\nulldelimiterspace} {z_{30} }}} \right) = - \Delta z \times 1.33 \times 10^{ - 3}$ nm/µm. When Δ*z* = 50 µm, 5 µm, and 0.5 µm, the spectral resolution of the hologram is |Δ*λ*| = 0.0665, 0.00665 and 0.000665 nm, respectively. And, the corresponding spectral resolvance (*λ*/Δ*λ*) is 7.74 × 10^3^, 7.74 × 10^4^, and 7.74 × 10^5^, respectively. A resolvance of 7740 to 77,400 is in the medium to high resolution range and is a good range for determining stellar properties and radial velocities of stars in the Milky Way galaxy. A resolvance of 774,000 is in the super-resolution range for the detection of exoplanets by the radial velocity method. Noted that, to extract a selective bandwidth of light over a length Δz, a secondary optics can be used to diffract light away from the z-axis for an easy and full spectral analysis. A schematic of the approach by a diffracted optical element is illustrated in the inset of Fig. 7a.

Alternatively, a fiber or detector may be placed facing the *z* to collect starlight, such as shown in the photo of Fig. 6a*.* In this case, the minimum detector size *d* is set by the diameter of the Airy disk^13^ of our Fresnel hologram, i.e., *d* ≥ *d*_Airy_. For our Fresnel hologram, its diameter is *D* = 10 cm and the corresponding diameter of Airy disk is $d_{Airy} = 2.44 \times \frac{{\lambda z_{3} }}{D} = 2.44 \times \frac{{\lambda_{i} z_{30} }}{D} = 4.8$ µm. Interestingly, since *d*_Airy_ depends on intrinsic parameters such as *λ*_*i*_ and *z*_30_ and *D*, its value remains the same for all wavelengths or it is Δ*λ*-independent. Additionally, if we increase the diameter of our hologram to *D*' = 1 m, the corresponding Airy disk is reduced to $d^{\prime}_{Airy} = 2.44 \times \frac{{\lambda_{i} z_{30} }}{{D^{\prime}}} = \frac{{1.22\lambda_{i} }}{N.A.}\sim 1.22\lambda_{i} \approx 0.5$ µm. Here, the N.A. (Numerical Aperture) of holographic lens is close to 1 and the recording wavelength *λ*_i_ is 405 nm. It is this diffraction limit that determines the minimum *d* of a photodetector to be used and, therefore, the minimum Δ*z* for starlight collection.

According to the geometry shown in Fig. 7a, the amount of light collected over a diameter *d* (≡ Δ*y*) is equivalent to those light distributed over a length Δ*z* along the *z* axis. If the light incident angle is θ, we have $\Delta z = \frac{\Delta y}{{2 \times \tan \theta }}$. Here, $\theta = \tan^{ - 1} \left( {\frac{{\text{R}}}{{z_{30} }}} \right) = \tan^{ - 1} \left( {\frac{20}{{388}}} \right) = 3.0^{o}$. We may now re-examine the experimental configuration and spectral linewidth shown in Fig. 6a,b, respectively. Since the fiber has a diameter *d* = Δ*y* = 400 μm, the corresponding detecting length $\Delta z = \frac{\Delta y}{{2 \times \tan \theta }} = \frac{{400 \times 10^{ - 3} }}{2 \times 0.05} = 4$ mm. By Eq. (5), the predicted spectral linewidth $\left| {\Delta \lambda } \right| \cong \Delta z \times 1.33 \times 10^{ - 3} \;{{{\text{nm}}} \mathord{\left/ {\vphantom {{{\text{nm}}} {\mu {\text{m}}}}} \right. \kern-\nulldelimiterspace} {\mu {\text{m}}}} \cong 5.3\;{\text{nm}}$ at *z*_30_ = 388 mm. This finding agrees with the measured results shown in Fig. 6b within ~ 2 nm.

Separately, the Fresnel hologram may be utilized for a sensitive starlight detection. We will estimate the minimum detectable power of a micro-PMT detector and then examine the star light flux of alpha Lyrae, the 5th brightest star in the night sky. A typical micro-PMT has a noise level V_noise_ = 6 mV for a detector area A = 3 × 3mm^2^^18^. Its detectivity is such that a 1pW will lead to a signal level of V_signal_ = 15 mV. This gives a signal-to-noise ratio of 15 mV/6 mV = 2.5. Let us assume that this is an acceptable S/N ratio and 1pW is the minimum detectable power of such a PMT detector. For our hologram detecting system, incoming light may be concentrated to A_det_ ~ 500 × 500 μm^2^, and so the PMT detector size may be reduced accordingly. Given that a detector's noise voltage, such as the Johnson and shot noises, is proportional to the square root of the optically active area^19^, its noise voltage becomes V′_noise_ = 1 mV when its area is reduced by a factor 36 times to A_det_ ~ 500 × 500 µm^2^. So, the minimum detectable voltage is also reduced and given by V′_signal_ = V′_noise_ × 2.5 = 2.5 mV. The minimum detectable power of a reduced area micro-PMT is ${{1\;{\text{pW}}} \mathord{\left/ {\vphantom {{1\;{\text{pW}}} {\sqrt {36} }}} \right. \kern-\nulldelimiterspace} {\sqrt {36} }} \approx 1.6 \times 10^{ - 13} \;{\text{W}}$. If we further assume that our 10 × 10 cm^2^ Fresnel hologram as a 30% diffraction efficiency, the minimum detectable power of a reduced area PMT is ${\text{P}}_{\min } \approx 5.3 \times 10^{ - 13} \;{\text{W}}$.

We now examine the star light flux. A star with apparent magnitude V = 0 and zero color which is approximately the apparent magnitude and color of alpha Lyrae, the 5th brightest star in the night sky, has a flux of 37.4 × 10^–12^ W/m^2^/nm^20,21^ over the wavelength range of the V filter. The V filter has a spectral width of 88 nm and is centered at *λ* = 545 nm. Therefore, for a hologram that is 10 × 10 cm^2^, the flux of a 0th magnitude stars with zero color is 37.4 × 10^–14^ W/nm. From Eq. (5), the spectral width for a detector size of *d* = 500 µm and Δ*z* = 5 mm is Δ*λ* = 6.65 nm. So, the starlight power is (37.4 × 10^–14^ W/nm) × (6.65 nm) = 2.48 × 10^–12^ W. It is 4.5 times larger than the minimum detectable power of our Fresnel hologram with 30% diffraction efficiency. As a result, the Fresnel hologram holds great promise for a sensitive detection for space exoplanet telescope.

## Conclusion

In summary, we present the design of an unique annular Fresnel hologram based on the interference of two spherical waves. Through an analytical design, we found that the focal-length of our hologram can be tailored by tuning the radius-of-curvature of the two spherical waves. The grating period of the Fresnel hologram decreases monotonically from the center to the outer region and brings the incoming star-light to focus. Experimentally, we have successfully recorded our Fresnel hologram based on spherically symmetrical waves, with a focal-length by design. Furthermore, we have demonstrated a large on-axis dispersive behavior of an incoming, collimated light wave. This work represents the first successful implementation of a scale model optical system for realizing a telescope Fresnel hologram, which can achieve a spectral dispersion of *λ* = 440–705 nm over an on-axis distance of 190 mm. Therefore, our proposed DUET can simultaneously perform high resolution spectroscopy, high detection sensitivity and have a low areal mass for space exploration.

## Methods

### Reconstruction of the on-axis hologram with two spherical recording waves

Let us consider a plane wave *U*_P_ illuminating the hologram at normal incidence and with a wavelength *λ*, Fig. 2a. The hologram is recorded using two spherical waves of wavelength *λ*_i_, placed at *z*_R_ and *z*_O_, respectively. The recorded, constructive interference regions attenuate the illuminating (reading) light, so the transmittance of the hologram^22,23^ is:

6 $$t_{A} (x,y,z = 0) = t_{0} - \kappa \times I\left( {x,y,z = 0} \right)$$

Here, *t*_0_ and κ are both constants and their values dependent on the holographic material chosen. Accordingly, the transmitted wave propagating through the hologram, *U*_t_(x, y, z = 0), is given by:

7 $$\begin{aligned} U_{t} (x,y,z = 0) & = U_{P} \times t_{A} (x,y,z = 0) = U_{P} \times [t_{0} - \kappa \times I(x,y)] \\ & = U_{P} \times [t_{0} - \kappa \left| {U_{R} + U_{O} } \right|^{2} ] \\ & = U_{P} (t_{0} - \kappa \left| {U_{R} } \right|^{2} ) - \kappa U_{P} \left| {U_{O} } \right|^{2} - \kappa U_{P} U_{R}^{*} U_{O} - \kappa U_{P} U_{R} U_{O}^{*} \\ & = U_{1} + U_{2} + U_{3} + U_{4} \\ \end{aligned}$$

Here, *U*_1_ and *U*_2_ are both constants of (*x*, *y*). They are plane waves that directly transmit through the hologram. *U*_3_ and *U*_4_ are complex conjugate terms. They represent the two diffracted spherical waves. *U*_3_ is a real image and *U*_4_ a virtual image. *U*_3_ may be further computed to be:

8 $$\begin{aligned} U_{3} (x,y,z = 0) & = - \kappa U_{P} U_{R}^{*} U_{O} = - \kappa A_{P} A_{R}^{*} A_{O} e^{{i\left( {\phi_{A} + \phi_{O} - \phi_{R} } \right)}} = A_{3} e^{{i\phi_{3} }} \\ \phi_{3} \equiv \phi_{P} + \phi_{O} - \phi_{R} & = - \frac{\pi }{\lambda }\left[ {\left( {x^{2} + y^{2} } \right)\left( {\frac{1}{{z_{O} }}\frac{\lambda }{{\lambda_{i} }} - \frac{1}{{z_{R} }}\frac{\lambda }{{\lambda_{i} }}} \right) - 2x\left( {\frac{{x_{O} }}{{z_{O} }}\frac{\lambda }{{\lambda_{i} }} - \frac{{x_{R} }}{{z_{R} }}\frac{\lambda }{{\lambda_{i} }}} \right) - 2y\left( {\frac{{y_{O} }}{{z_{O} }}\frac{\lambda }{{\lambda_{i} }} - \frac{{y_{R} }}{{z_{R} }}\frac{\lambda }{{\lambda_{i} }}} \right)} \right] \\ \end{aligned}$$

When (*x*_R_, *y*_R_) = (0, 0) and (*x*_O_, *y*_O_) = (0, 0), *ϕ*_3_ can be simplified as:

9 $$\therefore \phi_{3} \equiv \phi_{P} + \phi_{O} - \phi_{R} = - \frac{\pi }{\lambda }\left[ {\left( {x^{2} + y^{2} } \right)\left( {\frac{1}{{z_{O} }}\frac{\lambda }{{\lambda_{i} }} - \frac{1}{{z_{R} }}\frac{\lambda }{{\lambda_{i} }}} \right)} \right]$$

Let us demand that the wave represented by *U*_3_ is to produce a point image at *z* > 0, so it must be a spherical wave originated from a point source at (*x*_3_, *y*_3_, *z*_3_). Similar to Eq. (2), *U*_3_ may also be written as:

10 $$\begin{aligned} U_{3} & = \left( {A_{3} /z_{3} } \right)e^{{ik\sqrt {(x - x_{3} )^{2} + (y - y_{3} )^{2} + (z - z_{3} )^{2} } }} \approx \left( {A_{3} /z_{3} } \right)e^{{ik\phi_{3} }} \\ \phi_{3} & \equiv - \frac{\pi }{{\lambda z_{3} }}\left( {x^{2} + y^{2} - 2xx_{3} - 2yy_{3} } \right) \\ \end{aligned}$$

For the first order diffraction, we match and equate the phase factors *ϕ*_3_, between Eqs. (9) and (10). As a result, the focal point (*x*_3_, *y*_3_, *z*_3_) of the image is given by:

11 $$\begin{gathered} x_{3} = y_{3} = 0; \hfill \\ \frac{1}{{z_{3} }} = \left( {\frac{1}{{z_{R} }} - \frac{1}{{z_{O} }}} \right)\frac{\lambda }{{\lambda_{i} }}{\text{ or }}z_{3} \lambda = \left( {\frac{1}{{z_{R} }} - \frac{1}{{z_{O} }}} \right)^{ - 1} \lambda_{i} \hfill \\ \end{gathered}$$

## References

[1] Lin SY, Chow E, Hietala V, Villeneuve PR, Joannopoulos JD (1998). Experimental demonstration of guiding and bending of electromagnetic waves in a photonic crystal. Science.

[2] Lin SY, Hietala VM, Wang L, Jones ED (1996). A highly dispersive photonic band gap prism. Opt. Lett..

[3] Hsieh ML, Chen HY, Peng CT, Lin S-Y (2015). Lens-less bending and concentration of light by volume hologram. Opt. Commun..

[4] Hsieh ML, Chen HY, Lin S-Y (2014). Observation of a large diffraction efficiency and efficiency enhancement of PQ/PMMA materials. Opt. Commun..

[5] Barton M, Britten JA, Dixit SN, Summers LJ, Thomas IM, Rushford MC, Lu K, Hyde RA, Perry MD (2001). Fabrication of large-aperture lightweight diffractive lenses for use in space. Appl. Opt..

[6] Meinel AB, Meinel MP (2002). Parametric dependencies of high-diffraction-order achromatized aplanatic configurations that employ circular or crossed-linear diffractive optical elements. Appl. Opt..

[7] Meinel AB, Meinel MP (2002). Large membrane space optics: Imagery and aberrations of diffractive and holographic achromatized optical elements of high diffraction order. Opt. Eng..

[8] MacEwen HA, Breckinridge JB (2013). Large diffractive/refractive apertures for space and airborne telescopes. Proc. SPIE Sens. Syst. Space Appl..

[9] Andersen G (2005). Large optical photon sieve. Opt. Lett..

[10] Ditto TD, Friedman JF, Content DA (2011). Astronomical telescope with holographic primary objective. Proc. SPIE.

[11] Ditto TD, Hsieh M-L, Newberg HJ, Swordy L, Hoyt R, Cushing J (2020). Optical space telescope without mirrors. Proc. SPIE.

[12] Sweatt WC (1977). Describing holographic optical elements as lenses. J. Opt. Soc. Am..

[13] Benton, S. A. & Bove, V. M. *Holographic Imaging*, Chap. 9 (Wiley, 2008) ISBN: 978-0-470-22412-0.

[14] Bennett, C. A. *Principle of Physical Optics*, Chap. 4, 125, Chap. 6, 321, Chap. 6, 295 (Wiley, 2008).

[15] Zemax® 13 is a commercially available optical design program from Radiant Zemax LLC 1990–2013. https://www.radiantzemax.com.

[16] A description of the Cassegrain Reflecting telescope. https://en.wikipedia.org/wiki/Reflecting_telescope.

[17] Schubert EF (2003). Light Emitting Diodes.

[18] Hamamatsu’s product on “Micro-PMT photodetector”. https://www.hamamatsu.com/us/en/product/optical-sensors/pmt/micro-pmt/micro-pmt-package/index.html.

[19] Dereniak EL, Boreman GD (1997). Infrared Detectors and Systems.

[20] Bessell MS (1999). Spectrophotometry: Revised standards and techniques. Pub. Astron. Soc. Pac..

[21] Bessell MS (2005). Standard photometric systems. Annu. Rev. Astron. Astrophys..

[22] Collier RJ, Burckhardt CB, Lin LH (1971). Optical Holography.

[23] Kogelnik H (1969). Coupled wave theory for thick hologram gratings. Bell Syst. Tech. J..

